# Botany

**Published:** 1892-11

**Authors:** 


BOTANY—A concise Manual for Students of Medicine and Science. By Alex Johnstone, F. G. S., Lecturer on Botany, School of Medicine, Edinburgh. 164 illustrations and a series of floral diagrams; pp. 260. Price—. New York. D. Appleton & Co., 1891.

This little work is concise and practical, and certainly of much value to the busy student as he follows the more elaborate work of the professor of botany. This branch is much neglected in medical education in this country, sorry to say. B.

